# How prevalent is a cancer-protective lifestyle? Adherence to the 2018 World Cancer Research Fund/American Institute for Cancer Research cancer prevention recommendations in Switzerland

**DOI:** 10.1017/S0007114522003968

**Published:** 2023-09-14

**Authors:** Nena Karavasiloglou, Giulia Pestoni, Sarah Theresa Pannen, Katja Angela Schönenberger, Tilman Kuhn, Sabine Rohrmann

**Affiliations:** 1 Division of Chronic Disease Epidemiology, Epidemiology, Biostatistics and Prevention Institute, University of Zurich, Hirschengraben 84, CH-8001 Zurich, Switzerland; 2 Nutrition Group, Health Department, Swiss Distance University of Applied Sciences, Zurich, Switzerland; 3 Department of Diabetes, Endocrinology, Nutritional Medicine and Metabolism, Inselspital, Bern University Hospital, University of Bern, Bern, Switzerland; 4 Division of Clinical Pharmacy and Epidemiology, Department of Pharmaceutical Sciences, University of Basel, Basel, Switzerland; 5 Institute for Global Food Security, Queen’s University Belfast, Belfast, Northern Ireland; 6 Heidelberg Institute of Global Health, University of Heidelberg, Heidelberg, Germany

**Keywords:** Cancer, Prevention, Guidelines, Lifestyle, Survey, Population-based

## Abstract

Population monitoring of lifestyle behaviours that are crucial as risk and protective factors for major chronic diseases is vital for the identification of priority areas for public health. In this study, we aimed to investigate the prevalence of adherence to the World Cancer Research Fund/American Institute for Cancer Research (WCRF/AICR) cancer prevention recommendations in Switzerland, overall and by selected sociodemographic and lifestyle characteristics. Data from the population-based, cross-sectional survey menuCH were used. We constructed a score reflecting adherence to the 2018 WCRF/AICR cancer prevention recommendations. Multinomial logistic regression models were fitted to investigate the association of sociodemographic and lifestyle characteristics with the level of adherence to the WCRF/AICR cancer prevention recommendations. The least frequently met cancer prevention recommendations were the ones on fibre intake (met by 13·7 %), red and processed meat (25·4 %), and ultra-processed food (33·3 %) consumption, while the recommendation on physical activity was met by almost 80 %. Women and individuals with tertiary education were more likely to have a score of ≥ 5 (as a reflection of adherence to the cancer prevention recommendations), compared with men or those who completed secondary education, respectively. Current smokers were less likely to have a score of ≥ 5, compared with never smokers. A high proportion of the population in Switzerland was found to not adhere closely to the WCRF/AICR cancer prevention recommendations. Differences were detected based on sociodemographic characteristics. Education and policy actions are needed to facilitate the adoption of a cancer-protective lifestyle.

Causal associations between dietary factors, body fatness and physical activity with cancer risk highlight the importance of healthy lifestyle in cancer prevention. The existing body of evidence has led many professional organisations and scientific societies to publish guidelines on diet and physical activity to help individuals make healthier choices. Most of these guidelines are created through the lens of overall well-being and chronic disease prevention. However, two bodies, the World Cancer Research Fund (WCRF) and the American Institute for Cancer Research (AICR), have released recommendations that, although instrumental for chronic disease prevention in general, show a particular focus on cancer^(1)^.

When focusing on individual lifestyle factors and their association with health outcomes, the potential synergistic or antagonistic effects between them, which could modify the overall association between lifestyle and disease risk, may be overlooked. To overcome this, a commonly used approach in epidemiological studies combines multiple lifestyle factors into scores. These factors can be independently chosen or be part of health-promoting nutrition/lifestyle recommendations from health organisations. The scores are then used when analysing the association with health outcomes^(2–5)^. Indeed, the cancer prevention recommendations issued by the WCRF/AICR have been widely used and linked to a lower risk of total cancer and various cancer types^(6–10)^.

To monitor population health, and to help inform public health priorities, many countries and scientific institutions assess lifestyle factors in representative health surveys^(11)^. Based on such survey data, trends in lifestyle exposures over time and subgroups of the population, for example defined by geographical areas or socio-economic characteristics, can be identified. In this study, using a nationally representative study population, we aimed to investigate the prevalence of adherence to the 2018 WCRF/AICR cancer prevention recommendations in Switzerland, overall and by selected sociodemographic and lifestyle characteristics, so as to provide a better understanding of the prevalence of cancer-protective behaviours in Switzerland and potentially inform future policy decisions aimed at cancer prevention.

## Methods

### Data and study population

The National Nutrition Survey menuCH is a population-based, cross-sectional survey that was conducted in Switzerland in 2014 and 2015. The study population consisted of a stratified random sample of the non-institutionalised residents of Switzerland aged 18 to 75 years provided by the Federal Office of Statistics. The survey was designed to be representative of the major geographical areas of Switzerland, the three main linguistic regions, and predefined age groups (18–29, 30–44, 45–59 and 60–75 years old). A detailed flow diagram of study participation has been published^(12)^. Overall, 2086 people took part in the menuCH. This study was conducted according to the guidelines laid down in the Declaration of Helsinki. All procedures involving human subjects were approved by the corresponding regional ethics committees and the lead committee in Lausanne (Protocol 26/13, approved on 12 February 2013). Written informed consent was obtained from all participants. The survey was registered (International Standard Randomised Controlled Trial Number (ISRCTN): ISRCTN16778734). Only participants who replied to both 24-h dietary recall interviews were included in our study (*n* 2057).

### Sociodemographic, dietary and lifestyle variables

#### Body weight and waist circumference

Information on sociodemographic and lifestyle variables was collected through questionnaires^(12)^. Body weight, height and waist circumference were measured by trained personnel, as described elsewhere^(13)^. For pregnant and lactating women or when measuring was not possible, the self-reported values for body weight (*n* 34) and height (*n* 7) were used. In pregnant and lactating women, the self-reported values reflected their pre-gestational weight. BMI (kg/m^2^) was calculated and categorised according to guidelines of the WHO^(14)^.

#### Physical activity

Physical activity was assessed via the short form of the International Physical Activity Questionnaire (IPAQ)^(15,16)^.

#### Dietary intake

Dietary and alcohol intake were assessed through two non-consecutive 24-h dietary recall interviews, as described previously^(13)^. Briefly, the first 24-h dietary recall took place in person and the second over the phone, approximately 2 to 6 weeks after the first, equally distributed across all weekdays and seasons. A book with 119 series of five to six graduated portion size pictures and a set of about 60 household measures^(17)^ were used to help assess the amount of food consumed. Food consumption of participants was recorded using the trilingual Swiss version (0.2014.02.27) of the software GloboDiet® (formerly EPIC-Soft®, International Agency for Research on Cancer IARC, Lyon, France^(18,19)^, adapted for Switzerland by the Federal Food Safety and Veterinary Office, Bern, Switzerland). Data cleaning was done after the completion of data collection using an updated version of the GloboDiet® software (0.2015.09.28).

#### Dietary supplements

The use of dietary supplements was assessed via a self-administered questionnaire. Participants were asked whether they took vitamins, minerals, combined supplements (i.e. containing both vitamins and minerals), or other dietary supplements in the past month, and whether these were prescribed by a medical professional or not.

### World Cancer Research Fund/American Institute for Cancer Research cancer prevention recommendations

We constructed a score reflecting adherence to the WCRF/AICR cancer prevention recommendations released in 2018^(1)^. Of the eight recommendations included in the recently proposed standardised scoring system^(20,21)^, we used those reflecting healthy body weight, physical activity, consumption of plant-based foods, highly processed foods, sugar-sweetened drinks, red and processed meat, and alcohol for the construction of the score in our study. Since information on supplement use was collected in menuCH, we constructed an additional score including supplement use (no supplement use or supplements prescribed by doctors *v*. self-medication) as a binary variable (1 *v*. 0 points, respectively) in sensitivity analyses. The WCRF/AICR recommendation on breast-feeding and the specific recommendation for cancer survivors were not used in our study, as in the standardised score.

We based our scoring as much as possible on the standardised scoring system proposed recently^(20,21)^. Detailed information on the operationalisation of the cancer prevention recommendations in the present study can be found in Supplementary Table 1. Briefly, the different recommendations were scored based on how closely survey responders followed them (0 points for no adherence, 0·5 points for moderate adherence and 1 point for high adherence). Since the dietary assessment in menuCH was conducted via two 24-h dietary recall interviews, the mean consumption of both interviews was considered for the diet-related cancer prevention recommendations. The score was constructed such that each recommendation contributed equally to the total lifestyle score, as proposed in the standardised scoring system^(20,21)^. The score ranged from zero to seven, and higher scores reflected greater adherence to the cancer prevention recommendations. The score was additionally categorised into three groups: low adherence (< 3 points), moderate adherence (3–<5 points) and high adherence (≥ 5 points).

### Statistical analyses

Baseline characteristics of study participants were reported as survey-weighted proportions, overall and across the categories of adherence to the WCRF/AICR cancer prevention recommendations.

Multinomial logistic regression models were fitted to investigate the association of sociodemographic and lifestyle characteristics with the level of adherence to the WCRF/AICR cancer prevention recommendations. Two models were fitted: model 1 was adjusted for age, sex and mean energy intake, whereas model 2 was further adjusted for language region, civil status, nationality, education and smoking status.

Due to the number of missing values, particularly in physical activity (25·5 % missing), multivariate imputation by chained equations (m = 25) was performed. The variables imputed included physical activity, waist circumference (n_missing_ = 34; as described above), supplement intake (n_missing_ = 7), education (n_missing_ = 3) and smoking status (n_missing_ = 4). The previously described menuCH weighting strategy^(22)^ was applied to all analyses to account for survey non-response and sampling design.

Two sensitivity analyses were conducted. Firstly, a score including the seven previously described cancer prevention recommendations and supplement use (no supplement use or supplements prescribed by doctors *v*. self-medication) as a binary variable (1 *v*. 0 points, respectively; range of the total score 0–8) was used in the multinomial logistic regression models. Secondly, due to the high proportion of missing values in physical activity, we re-ran the previously described analyses excluding physical activity from the score.

The analyses were conducted using R software (version 4.1.1, R Foundation for Statistical Computing, Vienna, Austria). The package *survey* was used to report the weighted participant characteristics^(23)^. Multinomial logistic regression models were fitted using the package *nnet* ^(24)^, and multiple imputation was performed using the package *mice* ^(25)^.

## Results

The characteristics of the study participants overall and according to the level of adherence to the WCRF/AICR cancer prevention recommendations are shown in Table 1. Overall, only 25·7 % of the study participants had a score of ≥ 5 in our study. The vast majority (59·9 %) only scored ≥ 3, but below 5. A higher proportion of women had a score of ≥ 5, compared with men. A higher proportion of participants who had completed tertiary education and those who reported never smoking had a score of ≥ 5, compared with participants having completed only primary education and current smokers, respectively.

**Table 1.** Characteristics of the menuCH participants overall and by categories of a score that reflects adherence to the World Cancer Research Fund/American Institute for Cancer Research 2018 cancer prevention recommendations (*n* 2057)*

	Overall	0–<3 points	3–<5 points	5–7 points
Sex				
Female	50·2	10·1	54·4	35·5
Male	49·8	18·8	65·4	15·8
Linguistic region				
German-speaking	69·2	15·5	59·5	25·0
French-speaking	25·2	12·1	59·7	28·2
Italian-speaking	5·6	12·1	64·6	23·3
Age categories (years)				
18–29	18·8	12·7	62·3	22·7
30–44	29·9	15·0	62·3	22·7
45–59	29·8	14·3	61·1	24·6
60–75	21·6	15·3	56·4	28·3
Education				
Primary	4·7	11·7	66·4	21·9
Secondary	42·6	17·1	59·0	23·9
Tertiary	52·7	12·5	60·0	27·5
Smoking status				
Never	43·0	11·6	57·1	31·3
Former	33·6	15·3	59·1	25·6
Current	23·3	18·4	66·1	15·6
Self-reported health				
Very bad to medium	12·7	23·2	58·6	18·2
Good to very good	87·3	13·2	60·1	26·8
Currently on a weight loss diet				
Yes	5·4	14·3	60·2	25·6
No	94·6	14·4	59·9	25·7
Score reflecting adherence to the World Cancer Research Fund/American Institute for Cancer Research 2018 cancer prevention recommendations				
0–<3 points	14·4			
3–<5 points	59·9			
5–7 points	25·7			

* All results reported are weighted proportion. Weights accounted for sex, age, marital status, major area of Switzerland, nationality and household size.

**Fig. 1.**
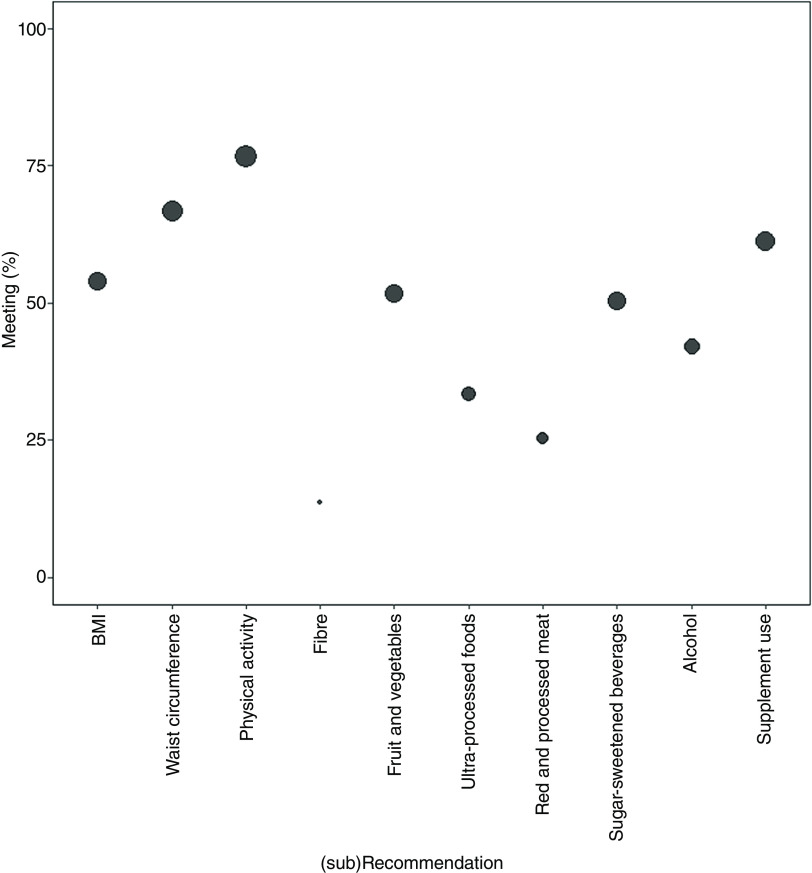
Weighted proportions of participants meeting each individual WCRF/AICR cancer prevention (sub-)recommendation. The size of the circles corresponds to the weighted proportions of participants fully meeting each recommendation. Survey weights were used to correct for non-response based on six sociodemographic parameters (i.e. age, sex, marital status, major area of Switzerland, nationality and household size).

The weighted proportions of menuCH responders meeting the individual WCRF/AICR cancer prevention recommendations (i.e. receiving the maximum points for the respective (sub-) recommendation) are shown in Fig. 1. The least frequently met cancer prevention recommendation was fibre intake (13·7 % participants meeting), followed by red and processed meat (25·4 %), and ultra-processed food (33·3 %) consumption. Almost 80 % of the menuCH participants were meeting the physical activity recommendation.

The results of multinomial logistic regression models investigating the association between personal characteristics and the level of adherence to the WCRF/AICR cancer prevention recommendations (as expressed via the score) in the fully adjusted model are presented in Table 2. Women were more likely than men to have a score of ≥ 5 (OR _≥ 5:_2·72, 95 % CI: 1·94, 3·81). Younger participants (18–29 years old) were more likely to have a score of ≥ 5, compared with those 30–44 years old (OR _≥__5:_1·88, 95 % CI: 1·12, 3·14). Similar results for women and younger participants were observed in the basic model adjusted for age, sex and energy intake (online Supplementary Table 2). Participants who had completed tertiary education were more likely to have a score of 3–<5 or ≥ 5, compared with those who completed secondary education (OR_3–<5 tertiary_: 1·32, 95 % CI: 1·01, 1·73; OR _≥__5 tertiary_:1·73, 95 % CI: 1·26, 2·37). Participants who were Swiss-binationals or non-Swiss were also more likely to have a higher score (OR _≥__5 binationals_:2·02, 95 % CI: 1·24, 3·29; OR _≥__5 non-Swiss_:1·52, 95 % CI: 1·03, 2·24), compared with Swiss participants. Current smokers were less likely to have a score of ≥ 5, compared with never smokers (OR _≥__5 current_: 0·42, 95 % CI: 0·28, 0·64). No significant differences were seen by linguistic region or civil status.

**Table 2.** Association between characteristics of the menuCH participants and categories of a score that reflects adherence to the World Cancer Research Fund/American Institute for Cancer Research 2018 cancer prevention recommendations (*n* 2057)*

	0–<3 points	3–<5 points		5–7 points	
		OR	95 % CI	OR	95 % CI
Sex					
Male	Ref.	Ref.		Ref.	
Female	Ref.	1·29	0·96, 1·73	2·72	1·94, 3·81
Age categories (years)					
18–29	Ref.	1·40	0·89, 2·19	1·88	1·12, 3·14
30–44	Ref.	Ref.		Ref.	
45–59	Ref.	1·13	0·80, 1·59	1·26	0·84, 1·90
60–75	Ref.	1·10	0·74 1·63	1·53	0·97, 2·43
Linguistic region					
German-speaking	Ref.	Ref.		Ref.	
French-speaking	Ref.	1·12	0·82, 1·54	1·15	0·81, 1·65
Italian-speaking	Ref.	1·38	0·74, 2·58	1·29	0·63, 2·64
Civil status					
Single	Ref.	Ref.		Ref.	
Married	Ref.	1·05	0·73, 1·51	0·82	0·54, 1·25
Divorced	Ref.	0·89	0·53, 1·48	0·89	0·50, 1·59
Other	Ref.	0·90	0·44, 1·84	1·25	0·58, 2·71
Nationality					
Swiss	Ref.	Ref.		Ref.	
Swiss binationals	Ref.	1·74	1·12, 2·70	2·02	1·24, 3·29
Non Swiss	Ref.	1·37	0·98, 1·91	1·52	1·03 2·24
Education					
Primary	Ref.	1·36	0·68, 2·73	1·30	0·58, 2·91
Secondary	Ref.	Ref.		Ref.	
Tertiary	Ref.	1·32	1·01, 1·73	1·73	1·26, 2·37
Smoking status					
Never	Ref.	Ref.		Ref.	
Former	Ref.	0·89	0·66, 1·21	0·72	0·51, 1·02
Current	Ref.	0·88	0·63, 1·22	0·42	0·28, 0·64

* Multinomial logistic regression model adjusted for sex, age categories (18–29, 30–44, 45–59 and 60–75 years old), mean energy intake, linguistic region, civil status, nationality, education and smoking status. Weights accounted for sex, age, marital status, major area of Switzerland, nationality, household size, as well as season and weekday of the dietary assessments.

Sensitivity analyses with a score including the additional cancer prevention recommendation on supplement use or with a score that excluded physical activity revealed largely similar results, with slight changes in the effect estimates (online Supplementary Table 3). Of note, the scores in the sensitivity analyses are not directly comparable to the score in the main results. While we used the same cut-offs for the categorical variable reflecting low, moderate and high scores, the range of the scores and the proportion of participants in the different levels of adherence differed considerably.

## Discussion

To our knowledge, this is the first study exploring the adherence to established cancer prevention recommendations and its sociodemographic determinants in Switzerland. Overall, less than one in three participants had a score of ≥ 5 reflecting higher adherence to the cancer prevention recommendation. We observed that the recommendations by the WCRF/AICR on fibre, red and processed meat, and ultra-processed food intake were the least frequently met recommendations among study participants. When looking at the association of sociodemographic and lifestyle characteristics with adherence to the cancer prevention recommendations, women and higher educated individuals were more likely to have higher scores, while the opposite was true for current smokers.

Our results are largely consistent with those reported in the literature. A study exploring opinions and behaviours related to healthy lifestyle and cancer among university students in Romania (data collected in 2017), reported high adherence to the physical activity WCRF cancer prevention recommendation, as well as a relatively high proportion of students within meeting the BMI cancer prevention recommendation. However, only 2·6 % of the participating students were meeting the fruit and vegetable recommendation and only about half were meeting the recommendation on meat consumption^(26)^. In contrast to our results that supported a high adherence to the physical activity recommendation, a Canadian cross-sectional study (data collected in 2008) reported only 19 % of participants meeting the WCRF/AICR cancer prevention recommendation on physical activity^(27)^. Low adherence to the physical activity recommendation was also reported by a previous study from Switzerland, using data collected before the mid-1990s^(28)^. The recommendations least likely to be met in a study from Sweden (data collected in 1997) were limiting the consumption of red and processed meat, limiting ‘fast food’ and other processed foods high in fat, starches or sugar and consuming plant foods. Similar to our results, half of the study population did not meet the healthy weight recommendation (51 %)^(6)^.

Our findings suggested that higher scores were more likely among women and higher educated individuals, and less likely among current smokers. Similar results were reported in a previous study from Switzerland using an earlier version of the WCRF/AICR cancer prevention recommendations^(28)^. These findings indicate that specific population groups may benefit from targeted interventions (i.e. men, people with lower education and current smokers) complementary to public health measures aiming at improving the lifestyle habits of the general population.

While we are the first to explore the determinants of adherence to the 2018 WCRF/AICR cancer prevention recommendations in Switzerland, previous studies have addressed the adherence to different health recommendations. Most of them report very low adherence to healthy eating recommendations, like the Dietary Guidelines for Americans (assessed via the Alternative Healthy Eating Index (AHEI)^(3)^) or the Swiss Food Pyramid food-based guidelines^(12,29–31)^. One previous study reported on the adherence to an earlier version of the WCRF/AICR cancer prevention recommendations in Switzerland, but the aim of the study was the association with mortality^(28)^. The low adherence to health recommendations has important public health implications, given the ageing population living in Switzerland and the rise in non-communicable diseases, including cancer. Policy actions (like the ones included in the NOURISHING and MOVING databases^(32)^), centred around healthy ageing, are needed to remove barriers to healthy eating and physical activity, and make healthier foods the easiest and most readily accessible option.

Our study has several strengths. The participants were drawn from a nationally representative stratified random sample. A weighting strategy was applied, correcting for changes in population structure due to non-response and for the uneven distribution of the dietary assessment across weekdays and seasons. The dietary intake of the menuCH participants was assessed by two non-consecutive 24-h dietary recall interviews using the reference software GloboDiet®, which has been shown to provide reliable estimates of the consumed nutrients and foods.

However, the present study also has some limitations. Recall bias and the potential under- or over-reporting in the 24-h dietary recall interviews cannot be excluded, as in any observational study based on self-reported dietary assessment. Study participants might have been more health-conscious than the general population, suggesting that the proportions of individuals in the general population not meeting the cancer prevention recommendations might be even higher. It should also be noted that it is unknown what proportion of the Swiss population is aware of the WCRF/AICR recommendations or other lifestyle recommendations. Thus, the term ‘adherence’ in this study was used to reflect the degree to which population-level lifestyle behaviours in the Switzerland are in agreement with the WCRF/AICR recommendations, rather than intentional adherence on the individual level.

In conclusion, a high proportion of the population in Switzerland was found to not meet the WCRF/AICR cancer prevention recommendations, with differences detected based on sociodemographic and lifestyle characteristics. Education and policy actions are needed to remove barriers to following a healthy lifestyle, support healthy ageing and assist the population in adhering to the cancer prevention recommendations.

## Supplementary material

For supplementary material accompanying this paper visit https://doi.org/10.1017/S0007114522003968. click here to view supplementary material
