# Supplementary material for: How prevalent is a cancer-protective lifestyle? Adherence to the 2018 World Cancer Research Fund/American Institute for Cancer Research cancer prevention recommendations in Switzerland
Source: Br J Nutr. 2022 Dec 21;130(5):904–10. doi: 10.1017/S0007114522003968 (PMC10404478; doi:10.1017/S0007114522003968)
Supplement: Supplementary file 1 [file S0007114522003968sup001.docx]

Supplementary Table 1. Operationalization of the 2018 World Cancer Research Fund/ American Institute for Cancer Research cancer prevention recommendations in the menuCH ^1^

| **World Cancer Research Fund/ American Institute for Cancer Research 2018 cancer prevention recommendations** | **Operationalization** | **Scoring** |
| --- | --- | --- |
| Be a healthy weight |  |  |
| Body mass index | Body mass index < 18.5 kg/m^2^ & Body mass index ≥ 30 kg/m^2^ | 0 |
|  | Body mass index ≥ 25.0 kg/m^2^ & Body mass index < 30.0 kg/m^2^ | 0.25 |
|  | Body mass index ≥ 18.5 kg/m^2^ & Body mass index < 25.0 kg/m^2^ | 0.5 |
| Waist circumference | Waist circumference ≥ 88 cm (for females) or 102 cm (for males) | 0 |
|  | 80 cm (for females) or 94 cm (for males) ≤ Waist circumference < 88 cm (for females) or 102 cm (for males) | 0.25 |
|  | Waist circumference < 80 cm (for females) or 94 cm (for males) | 0.5 |
| Be physically active |  |  |
|  | < 75 minutes/week moderate and/or vigorous physical activity | 0 |
|  | 75 minutes/week ≤ moderate and/or vigorous physical activity < 150 minutes/week | 0.5 |
|  | ≥ 150 minutes/week moderate and/or vigorous physical activity | 1 |
| Eat a diet rich in wholegrains, vegetables, fruit, and beans |  |  |
| Fiber intake | Fiber < 15.0 g /day | 0 |
|  | 15.0 g/day ≤ Fiber < 30.0 g/day | 0.25 |
|  | Fiber ≥ 30.0 g/day | 0.5 |
| Fruits and vegetables | Fruit and vegetables intake < 200 g/day | 0 |
|  | 200 g/day ≤ Fruit and vegetables intake < 400 g/day | 0.25 |
|  | Fruit and vegetables intake ≥ 400 g/day | 0.5 |
| Limit consumption of fast foods and other processed foods high in fat, starches, or sugars. Study wide tertiles of the adjusted NOVA classification in grams ^2^ |  |  |
|  | Tertile 1 ^2^ | 0 |
|  | Tertile 2 ^2^ | 0.5 |
|  | Tertile 3 ^2^ | 1 |
| Limit consumption of red and processed meat ^3^ |  |  |
|  | Red meat intake > 71.4 g/day or processed meat intake > 14 g/day | 0 |
|  | Red meat intake ≤ 71.4 g/day & 3 g/day < processed meat intake ≤ 14 g/day | 0.5 |
|  | Red meat intake ≤ 71.4 g/day & processed meat intake < 3g/day | 1 |
| Limit consumption of sugar-sweetened beverages |  |  |
|  | Sugar-sweetened beverage consumption > 250 g/day | 0 |
|  | 0 g/day ≤ Sugar-sweetened beverage consumption < 250 g/day | 0.5 |
|  | Sugar-sweetened beverage consumption = 0 g/day | 1 |
| Limit alcohol consumption |  |  |
|  | Alcohol consumption > 14 g/day (for females) or 28 g/day (for males) | 0 |
|  | Alcohol consumption < 14 g/day (for females) or 28 g/day (for males) | 0.5 |
|  | Alcohol consumption = 0 g/day | 1 |
|  |  |  |
| Sensitivity analysis |  |  |
| Do not use supplements for cancer prevention ^4^ |  |  |
|  | Reported supplement use based on the basis personal decisions | 0 |
|  | No reported supplement use/ Reported supplement use on the basis of recommendation by doctors | 1 |

^1^ For the dietary variables, we considered the mean consumption of the two 24-hour dietary recall interviews.
^2^ Food items already included in other components of the score (i.e., sugar sweetened beverages, red and processed meat, alcoholic drinks) were removed from the original NOVA ultra-processed food classification to create this adjusted NOVA variable. Although the proposed score by Shams-White et al., 2019 suggests tertiles based on the total energy from ultra-processed foods, we based our tertiles on grams from ultra-processed foods since we adjust the models for total energy intake. The study-wide tertiles of the adjusted NOVA classification correspond to consumption of < 111.7 gr (tertile 1); 111.7-236.6 g (tertile 2) and ≥ 236.6 g (tertile 3).
^3^ Since the red and processed meat recommendation was given in weekly format, we divided the proposed consumption by 7 to get the estimated daily consumption.
^4^ We assigned a lower score to participants who use supplements on the basis of personal decision because the World Cancer Research Fund explicitly mentions that the recommendation “Do not use supplements for cancer prevention” “applies to all doses and formulations of supplements, unless supplements have been advised by a qualified health professional who can assess potential risks and benefits” (<https://www.wcrf.org/diet-activity-and-cancer/cancer-prevention-recommendations/do-not-use-supplements-for-cancer-prevention/>).

Supplementary Table 2. Association between characteristics of the menuCH participants and a score that reflects adherence to the World Cancer Research Fund/ American Institute for Cancer Research 2018 cancer prevention recommendations (n = 2,057) *

|  |  | OR (95% CI) | |
| --- | --- | --- | --- |
|  | 0- <3 points | 3- <5 points | 5-7 points |
| Sex |  |  |  |
| Male | Ref. | Ref. | Ref. |
| Female | Ref. | 1.27 (0.95-1.69) | 2.89 (2.09-4.02) |
| Age categories, years |  |  |  |
| 18-29 | Ref. | 1.27 (0.86-1.88) | 1.73 (1.11-2.70) |
| 30-44 | Ref. | Ref. | Ref. |
| 45-59 | Ref. | 1.05 (0.76-1.45) | 1.11 (0.76-1.63) |
| 60-75 | Ref. | 0.91 (0.63-1.29) | 1.19 (0.79-1.79) |

* Multinomial logistic regression model adjusted for sex, age categories (18-29, 30-44, 45-59, 60-75 years old) and mean energy intake. Weights accounted for sex, age, marital status, major area of Switzerland, nationality, household size, as well as season and weekday of the dietary assessments. CI: Confidence interval, OR: Odds ratio.

Supplementary Table 3. Association between characteristics of the menuCH participants and a score that reflects adherence to the World Cancer Research Fund/ American Institute for Cancer Research 2018 cancer prevention recommendations (n = 2,057, using alternative scoring) *

|  | Score including supplement use | | | Score excluding physical activity | | |
| --- | --- | --- | --- | --- | --- | --- |
|  |  | OR (95% CI) | |  | OR (95% CI) | |
|  | 0- <3 points | 3- <5 points | 5-8 points | 0- <3 points | 3- <5 points | 5-6 points |
| Sex |  |  |  |  |  |  |
| Male | Ref. | Ref. | Ref. | Ref. | Ref. | Ref. |
| Female | Ref. | 1.46 (0.95-2.25) | 2.17 (1.41-3.36) | Ref. | 1.76 (1.42-2.18) | 2.26 (1.53-3.36) |
| Age categories, years |  |  |  |  |  |  |
| 18-29 | Ref. | 2.44 (1.26-4.72) | 2.27 (1.16-4.44) | Ref. | 1.02 (0.74-1.42) | 0.79 (0.43-1.44) |
| 30-44 | Ref. | Ref. | Ref. | Ref. | Ref. | Ref. |
| 45-59 | Ref. | 1.25 (0.77-2.03) | 1.47 (0.90-2.43) | Ref. | 1.03 (0.79-1.33) | 1.12 (0.69-1.81) |
| 60-75 | Ref. | 1.28 (0.71-2.29) | 1.72 (0.95-3.12) | Ref. | 1.47 (1.08-2.00) | 2.28 (1.34-3.88) |
| Linguistic region |  |  |  |  |  |  |
| German-speaking | Ref. | Ref. | Ref. | Ref. | Ref. | Ref. |
| French-speaking | Ref. | 1.23 (0.76-1.98) | 1.72 (1.06-2.79) | Ref. | 1.25 (1.00-1.58) | 0.78 (0.51-1.21) |
| Italian-speaking | Ref. | 1.51 (0.57-4.03) | 1.96 (0.73-5.25) | Ref. | 1.64 (1.04-2.59) | 1.22 (0.55-2.70) |
| Civil status |  |  |  |  |  |  |
| Single | Ref. | Ref. | Ref. | Ref. | Ref. | Ref. |
| Married | Ref. | 1.49 (0.91-2.46) | 1.10 (0.66-1.83) | Ref. | 0.80 (0.61-1.06) | 0.58 (0.36-0.93) |
| Divorced | Ref. | 1.06 (0.52-2.14) | 0.80 (0.39-1.63) | Ref. | 0.78 (0.53-1.15) | 0.60 (0.30-1.18) |
| Other | Ref. | 1.49 (0.46-4.84) | 1.97 (0.61-6.34) | Ref. | 1.28 (0.74-2.22) | 1.12 (0.48-2.63) |
| Nationality |  |  |  |  |  |  |
| Swiss | Ref. | Ref. | Ref. | Ref. | Ref. | Ref. |
| Swiss binationals | Ref. | 1.85 (0.91-3.79) | 2.13 (1.03-4.37) | Ref. | 1.27 (0.95-1.70) | 1.79 (1.08-2.96) |
| Non Swiss | Ref. | 0.94 (0.60-1.48) | 1.05 (0.66-1.67) | Ref. | 1.21 (0.94-1.55) | 1.90 (1.23-2.93) |
| Education |  |  |  |  |  |  |
| Primary | Ref. | 2.35 (0.59-9.32) | 2.99 (0.75-12.00) | Ref. | 1.09 (0.67-1.77) | 1.38 (0.57-3.32) |
| Secondary | Ref. | Ref. | Ref. | Ref. | Ref. | Ref. |
| Tertiary | Ref. | 1.02 (0.69-1.51) | 1.32 (0.89-1.97) | Ref. | 1.39 (1.13-1.71) | 1.93 (1.31-2.83) |
| Smoking status |  |  |  |  |  |  |
| Never | Ref. | Ref. | Ref. | Ref. | Ref. | Ref. |
| Former | Ref. | 1.69 (1.06-2.70) | 1.18 (0.74-1.90) | Ref. | 0.85 (0.68-1.07) | 0.46 (0.31-0.68) |
| Current | Ref. | 1.04 (0.66-1.64) | 0.60 (0.38-0.97) | Ref. | 0.80 (0.62-1.03) | 0.31 (0.18-0.54) |

* Multinomial logistic regression model adjusted for sex, age categories (18-29, 30-44, 45-59, 60-75 years old), mean energy intake, linguistic region, civil status, nationality, education, and smoking status. Weights accounted for sex, age, marital status, major area of Switzerland, nationality, and household size, as well as season and weekday of the dietary assessments. CI: Confidence interval, OR: Odds ratio.

The score including supplement use ranged from 0 to 8 points, while the score excluding physical activity ranger from 0 to 6 points.
